# Peer Support and Exclusive Breastfeeding Duration in Low and Middle-Income Countries: A Systematic Review and Meta-Analysis

**DOI:** 10.1371/journal.pone.0045143

**Published:** 2012-09-18

**Authors:** Christopher R. Sudfeld, Wafaie W. Fawzi, Chandrakant Lahariya

**Affiliations:** 1 Department of Epidemiology, Harvard School of Public Health, Boston, Massachusetts, United States of America; 2 Department of Nutrition, Harvard School of Public Health, Boston, Massachusetts, United States of America; 3 Department of Global Health and Population, Harvard School of Public Health, Boston, Massachusetts, United States of America; 4 Department of Community Medicine, G.R. Medical College, Gwalior, MP, India; University of Montreal, Canada

## Abstract

**Objective:**

To examine the effect of peer support on duration of exclusive breastfeeding (EBF) in low and middle-income countries (LMICs).

**Data Sources:**

Medline, EMBASE, and Cochrane Central Register for Controlled Trials were searched from inception to April 2012.

**Methods:**

Two authors independently searched, reviewed, and assessed the quality of randomized controlled trials utilizing peer support in LMICs. Meta-analysis and metaregression techniques were used to produce pooled relative risks and investigate sources of heterogeneity in the estimates.

**Results:**

Eleven randomized controlled trials conducted at 13 study sites met the inclusion criteria for systematic review. We noted significant differences in study populations, peer counselor training methods, peer visit schedule, and outcome ascertainment methods. Peer support significantly decreased the risk of discontinuing EBF as compared to control (RR: 0.71; 95% CI: 0.61–0.82; I^2^ = 92%). The effect of peer support was significantly reduced in settings with >10% community prevalence of formula feeding as compared to settings with <10% prevalence (p = 0.048). There was no evidence of effect modification by inclusion of low birth weight infants (p = 0.367) and no difference in the effect of peer support on EBF at 4 versus 6 months postpartum (p = 0.398).

**Conclusions:**

Peer support increases the duration of EBF in LMICs; however, the effect appears to be reduced in formula feeding cultures. Future studies are needed to determine the optimal timing of peer visits, how to best integrate peer support into packaged intervention strategies, and the effectiveness of supplemental interventions to peer support in formula feeding cultures.

## Introduction

Exclusive breastfeeding (EBF) has been identified as one of the most important preventive interventions for child survival [1], [2]. In 2001, the World Health Organization (WHO) recommended EBF for infants until 6 months of age [3]. This recommendation has been incorporated into national health policies and child survival programs in many low and middle income countries (LMICs) [4]–[6]. Nevertheless, low rates of EBF persist in LMICs and only 39% of infants are exclusively breastfed for 6 months (2000–2007) [7], [8]. Hospital based efforts like the Baby Friendly Health Initiative (BFHI) have only been partially successful in increasing EBF duration at the population level in LMICs where a large proportion of births occur at home [9], [10]. In addition, shortages of health workers at almost every level further limit the capacity of breastfeeding support at primary healthcare clinics in resource-limited settings [11], [12]. As a result, peer support has often been considered a viable alternative to counseling at health facilities [13].

Previous systematic reviews and meta-analyses examining the effect of peer support on EBF duration have been published [14]–[17]. The most recent meta-analysis by Jolly and colleagues found peer support had a significantly greater effect on EBF duration in low and middle income countries (RR for discontinuing EBF: 0.63 (95% CI: 0.52–0.78; I^2^ = 93.4%) as compared to high income countries (RR for discontinuing EBF: 0.90 (95% CI: 0.85–0.97; I^2^ = 82.4%) [17]. A possible explanation is peer support may be less effective in overcoming social preferences for infant formula feeding [18], [19]. For example, two randomized controlled trials conducted in Scotland and Hong Kong found no effect of peer support on EBF duration and both authors noted that cultural norms favoring bottle-feeding and a strong aversion to public breastfeeding were likely contributors to null findings [20], [21].

We hypothesize similar differences in availability and cultural preferences for infant formula may partially explain the high variability in the effectiveness of peer support on EBF duration in LMICs. An absence of infant formula commercial marketing, high cost infant formula, and negative attitudes of family members toward infant formula may create a context in some LMICs where peer support alone is very effective in overcoming barriers to EBF [17], [22]. We also hypothesize the effect of peer support on EBF duration may be greater for low birth weight infants, who may receive the greatest health benefits from EBF [2].

Here we present the results of a systematic review and meta-analysis limited to randomized controlled trials of peer support conducted in LMICs. We focus on examining the impact of formula feeding culture, inclusion of low birth weight infants, and infant age at the time of outcome assessment on the effect of peer support on EBF duration.

## Materials and Methods

### Systematic Review

We performed a systematic review of published randomized controlled trials following the criteria of the PRISMA statement [23]. Studies were identified from the following sources: Medline (from 1950 to April 2012), EMBASE (1966 to April 2012) and the Cochrane Central Register of Controlled Trials (1980 to April 2012). The following free test search strings were used (peer AND [exclusive breastfeeding OR breastfeeding]) and (community AND [exclusive breastfeeding OR breastfeeding]). Cited references from all published papers and relevant reviews were considered for inclusion. The ClincalTrials.gov website was also searched for randomized controlled trials that were registered but not yet published. All randomized controlled trials utilizing breastfeeding support interventions received a full article review. The search procedures were completed by two authors independently (CRS and CL).

The two main criteria for randomized controlled trials to be included in the final review database were i) include peer support as an intervention and ii) be conducted in a LMIC as defined by the World Bank [24]. We defined peer support as ‘the provision of emotional, appraisal and informational assistance by a created social network member who possesses experiential knowledge of a specific behavior or stressor and similar characteristics as the target population’ [25]. In this review peer counselors could also be classified as ‘lay’ since they received no formal medical, nursing, or nutritional training. All studies using various support methods (one-to-one vs. group support) and timing of visits (antenatal vs. postnatal) were included.

Standard information was extracted from each study fulfilling the inclusion criteria. The data sought included a description of study location, eligibility criteria, timing of peer support visits, peer counselor training protocols, any data on outcomes related to initiation or duration of breastfeeding, and also child health outcomes. The primary outcome of interest was EBF at the last study visit in the trial. We defined EBF using the WHO definition of maternal milk being the only food source with no other liquids or food given except medicines, minerals, and vitamins [26]. Data extraction was undertaken independently by two authors (CRS and CL) and entered in a standardized database. Any disagreements were adjudicated by a third reviewer (WWF).

**Figure 1:**
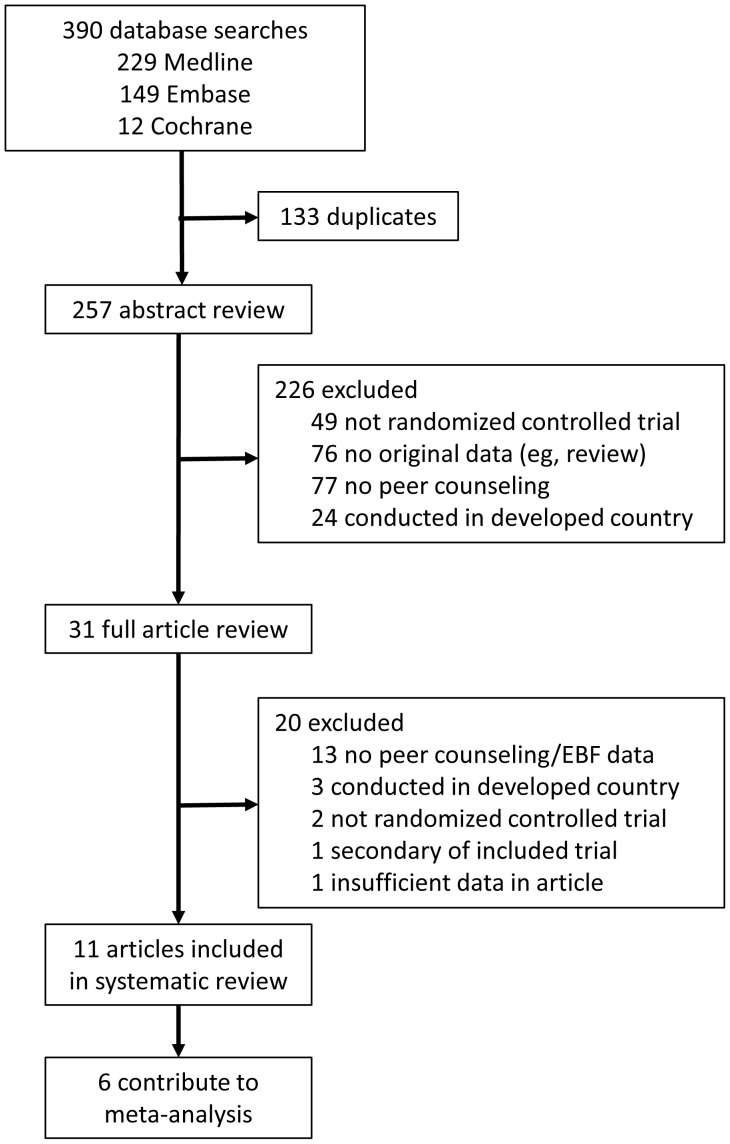
Flow diagram of included and excluded studies.

We also assessed the risk of bias for each trial based on the description of eligibility criteria, conduct of randomization, allocation concealment, similarity of groups at baseline, completeness of follow-up, interviewer blinding, use of an intention-to-treat analysis, and proper statistical adjustment for cluster randomized trials. Two authors independently classified the risk of bias for each trial as low, moderate, or high (CRS and CL) and any disagreements were adjudicated by a third reviewer (WWF).

### Meta-analysis

The primary outcome of EBF at the last trial visit was decided *a priori*. After an initial review of the trials, the authors determined that some trials should not be included in the final meta-analysis due to high risk of bias and some trials were unable to assess the effect of a peer counseling program on EBF duration past the neonatal period. Studies to be included in the final meta-analysis were determined by independent review of the trials and consensus of all authors (CRS, CL, and WWF). Study relative risks (RR) and 95% confidence intervals (CIs) were obtained from each study or calculated with event numbers extracted from the trial. One study only presented cluster adjusted estimates for risk differences and not relative risks [27]. In order to adjust for clustering the standard error for relative risk was inflated by the same correction factor as the risk difference [27]. Similar to Jolly et al., we used the relative risk of discontinuing EBF at the final study visit as the primary outcome [17]. This method allows peer support to have a greater absolute impact in settings where baseline EBF prevalence is low. Effect estimates from individual trials were pooled in random-effects models with inverse-variance weights to produce summary RRs and 95% CIs. The percentage of variability across studies attributable to heterogeneity was estimated with the I^2^ statistic [28]. The I^2^ index estimates the percentage of variability due to heterogeneity between trials rather than sampling error. Potential publication bias was assessed by funnel plots of the natural log of the RR vs. the standard error [29].

**Table 1:** Characteristics and results of included trials.

Trial	Location	Area	Study population	Intervention definition	Training	Comparison	EBF Assessment	Effect on Exclusive Breastfeeding	Child Health Outcomes
Agrasada (2005)^33^	Manila, Philippines	Urban	First-time mothers intending to breastfeed a low birth weight (<2500 g) singleton born between 37 and 42 wks	Home visits at days 3–5, 7–10, 21, 1.5 mo followed by monthly visits up to 5.5 month post-delivery.	40 hours of training from certified lactation counselor	1) No Support 2) Childcare support	Hospital interviews at 2 weeks, 4 weeks, and then monthly until 6 months	a) EBF 2 wk to 6 mo: PC 6.3 (3.53–11.3) times more likely to be exclusively fed at any time point [GEE analysis] b) EBF birth to 6 months: PC 32%, childcare group 3% and control 0%	a)No difference mean weight for age z-scores at 6 months. b) Decreased diarrhea in PC group (15%) vs. childcare (28.3%) and control (30.5%)
Aksu (2011)^40^	Aydın, Turkey	Urban	Singleton healthy infant ≥37 weeks. <2500 g, Apgar ≤7, congenital anomalies excluded.	Home visit 3 days after birth.	18-hour WHO/UNICEF breastfeeding support course	20–30 min. breastfeeding education from nurses	Household interviews at 2 weeks, 6 weeks, and at 6 months	a) EBF at 6 months: PC 43% and comp. 23% b) EBF at 6 weeks: PC 60% and comp. 33% c) EBF at 2 weeks: PC 67% and comp. 40%	
Arifeen (2009)^41^	Matlab, Bangladesh	Urban	Pregnant women in 20 government health facilities	IMCI home visits with village health workers and community nutrition promoters.	IMCI 2-day training course for feeding practices	Standard of care	Household census but method not directly stated	EBF 0–6 months: PC 76% and comp. 65%	a) U5 mortality rate was lower in IMCI than comp. areas (not significant )
Coutinho (2005)^34^	Pernambuco, Brazil	Urban	Singleton infants >2500 g without congenital anomalies or serious illness	Home visits on days 3, 7, 15, and 30, and then every 2 weeks during the 2nd month and once a month 3rd to 6th months.	20 hour WHO Breastfeeding Support course	Hospital-based intervention only (BFHI)	Household interviews at 10, 30, 60, 90, 120, 150, and 180 days	EBF at 6 months: PC 24% and comp. 3%	
Davies-Adetugbo (1997)^42^	Osun State, Nigeria	Rural	Mothers-infant pairs presenting to health facility for diarrhea.	Promoting EBF to 6 months of life at presentation to primary care clinic (day 0) and then on days 2 and 7 at home.	Adapted WHO/UNICEF 18-hour breastfeeding course	Standard of care	Home visit 7 and 21 by same peer supporters	a) EBF at day 21: PC 46% and comp. 8% b) EBF at day 7: PC 59% and comp. 6% (p<0.00001)	New episode of diarrhea by 21 days: PC 12% and comparison 22% (not significant)
Haider (2000)^27^	Dhaka, Bangladesh	Urban	Women aged 16–35 years excluded infants with weight <1.8 kg, or congenital anomalies	Visits at 48 h of delivery, one on day 5, one during days 10–14, and every two weeks for 2–5 months.	40 hours WHO breastfeeding support and King’s book	Routine Care	Household interviews at 72 hrs then monthly to 6 months	EBF at 6 months- PC 70% and comp. 6% (p<0·0001)	
Feldens (2007)^35^	Sao Leopoldo, Brazil	Urban	Singleton, full-term infant with >2500 g	Home visits within 10 days of birth and monthly up to 6 months	10 steps for feeding healthy infants’ +16 hours practicum	Routine assistance by pediatricians	Home visit at 6 and 12 months	a) EBF at 4 months: PC 35% and comp. 16% b) EBF at 1 month: PC 69% and comp. 49%	Decrease in odds of dental caries: OR 0.52 (0.27–0.97)
Jakobsen (1999)^43^	Bandim, Guinea Bissau	Mix	Registered pregnancies in Bandim health project	Advised EBF for 4–5 months at routine local health centre visits at 6, 10 and 14 weeks.	NA	Routine Care	Weekly Household interviews	a) EBF at 4 months: PC 4.1% and comp. 3.7%; RR 1.08 (0.69–1.46)	
Leite (2005)^36^	Fortaleza, Brazil	Urban	Singletonhealth infant **<**3000****g;	Home visits on the 5th day, 15th, 30th, 60th, 90th and 120^th^ days.	20 hrs Breastfeeding support: a training course	Routine Care	Household interviews at days 30 and 180	a) EBF at 4 months: PC 25% and comp. 19% (p = .044)	
Morrow (1999)^44^	San Pedro Martir, Mexico	Peri-urban	All pregnant women residing in the study area	1) 6 visit group: Mid and late pregnancy and weeks 1, 2, 4, and 8. 2) 3 visit group: late pregnancy, and week 1 and 2.	1 week classroom, 2 months in lactation clinics, and 6 months practice	Standard of care with no home-visits	Household interviews at 2, 4, and 6 weeks and at 2, 3 and 6 months	a)EBF at 3 months: 6 PC visits 67%, 3 PC visits 62%, and comparison 12%. b) Significantly more EBF overtime in PC groups vs controls p<0.0001 [GEE analysis]	Control infants significantly higher incidence of diarrhea to 3 months RR: 2.1 (90% CI: 1.11–4.04; p = 0.029)
Tylleskär (2011)^37^	Banfora, Burkina Faso	Rural	Singleton births without severe malformation	Home visits during the third trimesters and weeks 2, 4, 8, 16, and 20.	1 week WHO Breastfeeding support, HIV and Infant feeding crse.	Standard of care with no home-visits	Household interviews at 3 and 6 months	a) EBF at 6 months: i) 7-day recall -RR 7.53 (4.42–12.82) ii) 24-h recall -RR 3.33 (1.74–6.38)b) EBF at 3 months: i) 7-day recall - RR 3.27 (2.13–5.03) ii) 24-h recall RR 2.29 (1.33–3.92)	a) Diarrhea prevalence at 6 months by 2-week recall: PR - 0.83 (95% CI: 0.45–1.54)
Tylleskär (2011)^37^	Mbale District, Uganda	Rural	Singleton births without severe malformation	Home visits during the third trimester, within the first week, and then weeks 4, 7, and 10.	1 week WHO Breastfeeding support, HIV and Infant feeding crse.	Standard of care with no home-visits	Household interviews at 3 and 6 months	a) EBF at 6 months: i) 7-day recall - RR 4.66 (3.35–6.49) ii) 24-h recall :RR 3.83 (2.97–4.95) b) EBF at 3 months: i) 7-day recall - RR 2.30 (2.00–2.65) ii) 24-h recall RR 1.89 (1.70–2.11)	a) Diarrhea prevalence at 6 months by 2-week recall: PR - 0.82 (95% CI: 0.58–1.15)
Tylleskär (2011)^37^	Paarl, Umlazi & Rietvlei, South Africa	Peri-urban and Rural	Singleton births without severe malformation	Home visits during the third trimester, within the first week, and then weeks 4, 7, and 10.	1 week WHO Breastfeeding support, HIV and Infant feeding crse.	Standard of care with no home-visits	Household interviews at 3 and 6 months	a) EBF at 6 months: i) 7-day recall - RR 9.83 (1.40–69.14) ii) 24-h recall -RR 5.7 (1.33–24.26) b) EBF at 3 months: i) 7-day recall - RR 1.98 (1.30–3.02) ii)24-h recall -RR 1.72 (1.12–2.63)	a) Diarrhea prevalence at 6 months by 2-week recall: PR 1.31 (95% CI: 0.89–1.93)

We primarily utilized metaregression techniques to investigate sources of heterogeneity in the effect estimate of peer counseling on EBF duration. We limited metaregression analyses to three trial covariates we suspected may impact the effect of peer support *a priori* including: community prevalence of infant formula feeding, inclusion of low birth weight infants, and infant age at EBF assessment. We classified trials conducted in settings with >10% community prevalence of infant formula feeding (among infants <6 months) as having moderate to high levels of formula feeding. Trials conducted in settings with <10% community prevalence were defined as having low levels of formula feeding. The community prevalence of infant formula feeding was not reported for all trials. As a result, we utilized data from demographic health surveys (DHS) or community-based nutrition surveys to provide an estimate [30]–[32]. Trials conducted in Brazil, South Africa, and The Philippines were characterized by moderate to high levels of formula feeding [33], [34], [35], [36]. Trials conducted in Bangladesh, Burkina Faso, and Uganda were considered to have low levels of formula feeding [27], [37]. We also present meta-analysis pooled effect estimates and corresponding I^2^ index stratified by community prevalence of infant formula feeding, inclusion of low birth weight infants, and infant age at EBF assessment.

**Figure 2:**
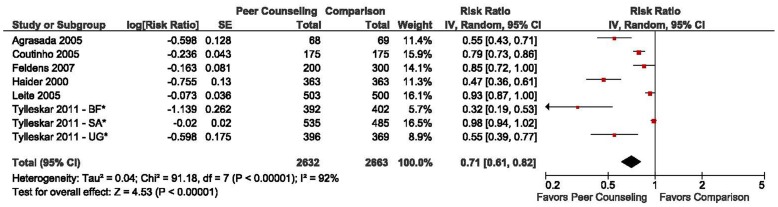
Pooled relative risk and 95% confidence intervals for the effect of peer support on discontinuing EBF. *Results from multicenter Tylleskär trial reported seperately. BF: Burkina Faso, SA: South Africa, UG: Uganda.

Metaregression was performed using the Stata metareg command, in which the natural logarithm of the relative risk was modeled as a linear function of the fixed trial-level covariate and a random trial specific intercept. Trials were weighted by the inverse of within-study and residual between-study variances [38]. We report results of univariate and multivariate metaregression analyses as regression coefficients (difference in log RR), 95% confidence intervals, and p-values. P-values less than 0.05 were regarded as statistically significant. Meta-analyses plots were created using RevMan 5.0 [39] and metaregression analyses employed Stata IC, version 10 (Statacorp, TX, USA).

### Ethics Statement

No ethical approval was necessary since the study was a review with no direct access to trial data. The decision to submit the article for publication was solely that of the authors and authors had access to all data.

**Table 2:** Results of univariate and multivariate random-effects metaregression.

Variable	No. Trial Sites* (Ref)	Relative Risk(95% CI)	I^2^	Univariatedifference inlog RR (95% CI)	p-value	Multivariatedifference inlog RR (95% CI)	p-value
***Community prevalence of formula feeding:***							
Moderate to High (>10%)	5 [33, 34, 35, 36, 37)	0.84 (0.74–0.95)	90%	0.59 (0.15–1.03)	0.016	0.59 (0.01–1.17)	0.048
Low (<10%)	3 [27], [37]	0.46 (0.36–0.59)	32%	Ref.	–	Ref.	–
***Inclusion of low birth weight infants:***							
Yes	2 [33], [36]	0.73 (0.43–1.21)	94%	0.12 (−0.67–0.90)	0.729	−.21 (−0.78–0.36)	0.367
No	6 [27], [34], [35], [37]	0.67 (0.55–0.83)	93%	Ref.	–	Ref.	–
***Infant age at assessment:***							
4 months	2 [35], [36])	0.92 (0.86–0.98)	3%	0.39 (−0.28–1.05)	0.204	0.19 (−0.37–0.76)	0.398
6 months	6 [27], [33], [34], [37]	0.61 (0.48–0.78)	94%	Ref.	–	Ref.	–

* Results of each study site for multicenter Tylleskär trial reported separately.

## Results

### Systematic Review

A broad literature search produced 390 articles for title and abstract review. A total of 31 of these studies were identified for full text review of which 11 randomized controlled trials of peer support conducted in low and middle income countries (LMICs) meet the inclusion criteria (Figure 1) [27], [34]–[37], [40]–[44]. The other 20 full review articles were excluded mainly for the reasons of not being a randomized controlled trial, the intervention consisted of clinically trained counselors, the study provided no original data (review) or the study was conducted in a country classified as high income by the World Bank (Table S2).

Table 1 summarizes the descriptive characteristics and results of the 11 included published RCTs that were conducted at 13 study sites in Latin America, South America, Southeast Asia, Eurasia, and sub-Saharan Africa. There were differences in the eligibility criteria for each of the studies. Five of the studies included all pregnant women [27], [37], [41], [43], [44], 3 enrolled women with singleton births of normal birth weight [34], [35], [40], 1 included mother-infant pairs presenting to a health facility for treatment of diarrhea [42], and two trials included only singleton births with low birth weights [33], [36]. The peer support schedule also differed between studies with the number of contacts ranging from 1 to 10 visits conducted during the antenatal period to six months postpartum. Additionally, the counselor training protocols varied and spanned from a single 18-hour training class to over 6 months of classroom and hands-on training [42], [44]. We also noted differences in the methods, frequency, and recall period (24-hour, 1 week, or 1 month) used to ascertain EBF duration. Despite the numerous differences in trial designs and populations, we present pooled and metaregression analyses in order to produce a more precise estimate of the effect of peer counseling on EBF duration and also investigate differences that may potentially modify the effect of peer counseling.

Six of the trials included data on child health outcomes [33], [35], [37], [41], [42], [44] (Table 1). Arifeen et al. was the only trial with data on child mortality and found reduced mortality that was not statistically significant in the trial arm including peer support [41]. Nevertheless, this trial assessed the multiple intervention WHO Integrated Management of Childhood Illness (IMCI) strategy and it is not possible to directly attribute any of the mortality effect to peer support. As for morbidity, Agrasada et al. and Morrow et al. both found significant reductions in incidence of diarrhea with peer support [33], [44]. On the other hand, there was no impact of peer support on prevalence of diarrhea at all three trial sites in the Tylleskär et al. study [37].

The quality of the RCTs was evaluated based on the authors’ judgment of bias risk. (Table S1). Seven of the trials were assessed to have low risk of bias [33]–[37], [44], [42], three studies were at moderate risk, [27], [40], [42] and one study was cited as high risk [43].

### Meta-analysis

Six trials conducted at 8 study sites contributed to the meta-analysis [27], [33]–[37]. These trials included a total of 5495 participants and the pooled relative risk of discontinuing EBF by the last study follow-up was 0.71 (95% CI: 0.61–0.82) for peer support versus control (Figure 2). This estimate was characterized by significant heterogeneity (I^2^ = 92%). We decided to not include the trial by Aksu et al. since the study provided peer support at only one visit 3 days postpartum and all other trials utilized greater than 5 visits [40]. The study by Morrow et al. was excluded because mothers who introduced supplementary feeding and then returned to EBF were classified as exclusive breastfeeders, which does not meet the WHO definition of EBF [44]. We also excluded the Jakobsen et al. trial since peer support was offered at immunization clinic visits and the study was noted to have high risk of bias [43]. The Arifeen et al. study was excluded since breastfeeding outcomes were assessed cross-sectionally for infants 0–6 months and results by infant age are not available [41]. We decided to exclude the Davies-Adetugbo trial since EBF was only assessed at 21 days postpartum [42].

We then utilized metaregression techniques to investigate community prevalence of formula feeding, inclusion of low birth weight infants, and infant age at EBF assessment as sources of heterogeneity in the effect estimate of peer support on EBF duration. Table 2 shows the results of univariate and multivariate metaregression analyses, along with stratified meta-analysis results for each of these variables. In univariate analyses the only statistically significant modifier was community prevalence of formula feeding (difference in log RR: 0.59; 95% CI: 0.15–1.03; p = 0.016). The relative risk of discontinuing EBF for peer support versus control was 0.46 (95% CI: 0.36–0.59) in settings with low levels of formula feeding (<10% community prevalence) as compared to 0.84 (95% CI: 0.74–0.95) in settings with moderate to high levels of formula feeding (>10% community prevalence). There was low to moderate heterogeneity in the effect estimate restricted to trials conducted in settings with low levels of formula feeding (I^2^ = 32%), while high heterogeneity remained in the estimate for trials conducted in settings with moderate to high levels of formula feeding (I^2^ = 90%). In a multivariate metaregression analysis, level of formula feeding remained the only statistically significant variable (difference in log RR: 0.59; 95% CI: 0.01–1.17; p = 0.048). There was no significant difference in the effect of peer counseling on EBF duration by inclusion of low birth weight infants (p = 0.367) or by assessment of EBF at 4 versus 6 months postpartum (p = 0.398).

## Discussion

This systematic review identified 11 randomized controlled trials examining the effect of peer support on EBF duration in LMICs [27], [34]–[37], [40]–[44]. We noted considerable differences, which have the potential to modify the effect of peer counseling, in study populations, peer counselor training protocols, peer visit schedule, and outcome ascertainment methods between trials. After the initial review of the database, the authors decided 5 of the trials should be excluded from the final meta-analysis due to high risk of bias and trial designs that were unable to assess the effect of a peer counseling program on EBF duration past the neonatal period. As a result, the authors may have induced bias through use of exclusion criteria created after the initial review; however, we tried to minimize this potential bias through independent reviews of each trial and agreement of all authors on the trials to be included in the meta-analysis. The meta-analysis of 6 trials conducted at 8 study sites found mothers with peer support were approximately 30% less likely to discontinue EBF at the final trial visit as compared to control mothers [27], [33]–[37]. Nevertheless, the I^2^ index for this estimate was 92%, which indicates a very high proportion of the variability in the analysis was due to differences between trials rather than sampling error.

In order to investigate sources of this heterogeneity, we performed metaregression analyses. A metaregression analysis determined the effect of peer counseling on EBF duration was significantly reduced in settings where the community prevalence of infant formula feeding was moderate to high (>10% community prevalence) as compared to settings where the level of formula feeding was low (<10% community prevalence). Additionally, the I^2^ for a meta-analysis restricted to trials conducted settings with low community prevalence of formula feeding was reduced to 32%, but high heterogeneity remained in the estimate for trials with moderate to high prevalence of infant formula feeding (I^2^ = 90%). The best illustration of effect modification by prevalence of formula feeding is the Tylleskär trial [37]. In this multicenter trial, a peer support intervention was found to significantly lengthen the duration of EBF at the Burkina Faso and Uganda trial sites, but there was no effect at the South Africa trial site. Tylleskär and colleagues suggest the availability of free infant formula to HIV-infected mothers and uncontrolled marketing of commercial formulas in South Africa resulted in communication of mixed messages on the optimal length of EBF. In contrast, there are no government formula programs and commercial marketing is minimal in Burkina Faso and Uganda where peer support was highly effective in increasing EBF duration to 6 months. Accordingly, taking in the cultural context and uniformity of EBF messages is essential when planning a peer support program. Supplemental interventions to peer support may be needed in populations where infant formula is culturally favorable.

The effect of peer support on child health was not clear. Peer support was found to significantly decrease the incidence of diarrhea in two trials, but there was no effect on diarrhea at all three Tylleskär trial sites [33], [37], [44]. The varying effect of peer support on diarrhea morbidity may be due differences in the method of infant feeding utilized by mothers who terminate EBF. Studies have found infants who predominantly breastfeed have only slight increases in diarrhea incidence as compared to infants who exclusively breastfeed [45]. As a result, trials conducted in communities where predominant breastfeeding is the principal breastfeeding alternative to EBF may not have adequate power to detect small differences in the incidence of diarrhea. Additional analyses and synthesis of trial data taking in account alternative breastfeeding methods may provide valuable insight into the impact of peer support on diarrhea.

Two studies included low birth weight infants and these trials found the strongest effect of peer counseling on EBF duration [33], [40]. The metaregression analysis did not find a statistically significant difference in the effect of peer support by inclusion versus exclusion of low birth weight infants; however, due to the small number of trials we may have had limited statistical power. Consequently, we may be underestimating the effect of peer support for low birth weight infants who may have the greatest health benefits from EBF [2].

Synthesis of trial results was difficult due to heterogeneity in study populations, training methods, and timing of peer counselor visits. The Jolly et al. review found the effect of peer support on EBF duration was significantly greater in trials with five or more planned visits [17]. We were unable to assess the impact of the number visits on the effect of peer support in our review, since only one study utilized less than 5 planned visits [40]. Accordingly, trials comparing the effectiveness of peer support interventions with different intensity and timing of peer support are needed to inform program planning. High intensity with many peer visits may not be financially viable in many resource-limited settings. There is also little data on the effect of peer support when integrated into a packaged maternal and child health (MCH) intervention. Arifeen et al. found the IMCI strategy, which included peer support, significantly increased the duration of EBF as compared to standard of care [41]. Nevertheless, there was some indication the effect of peer support in the Arifeen et al. trial was reduced compared to trials only communicating EBF messages. Furthermore, the effectiveness of peer support for HIV-infected mothers who are indicated for EBF when replacement feeding is not acceptable, feasible, affordable, sustainable, and safe (AFASS) has not been studied [46].

Peer support can increase the duration of EBF in LMICs. Countries pursuing Millennium Development Goal 4 should strongly consider including breastfeeding peer support in MCH programs. In order to maximize the potential benefits of peer support, studies are needed to determine the optimal timing and spacing of counselor visits, how to best integrate EBF messages into packaged MCH interventions, and the cost effectiveness of these strategies taking in account varying baseline rates of EBF. Studies are also need to determine if supplemental interventions can increase the effectiveness of peer support in LMICs with formula feeding cultures.

## Supporting Information

Table S1 **Bias assessment.**
(DOCX)

Table S2 **Studies excluded from the systematic review and meta-analysis.**
(DOCX)
