# Supplementary material for: Peer Support and Exclusive Breastfeeding Duration in Low and Middle-Income Countries: A Systematic Review and Meta-Analysis
Source: PLoS One. 2012 Sep 18;7(9):e45143. doi: 10.1371/journal.pone.0045143 (PMC3445598; doi:10.1371/journal.pone.0045143)
Supplement: Table S1 — Bias assessment. (DOCX) [file pone.0045143.s001.docx]

| Study | Complete description of eligibility criteria? | Proper conduct of randomization? | Allocation Concealment? | Similar groups at Baseline? | Loss to follow-up? Minimal (<10%), Moderate (10-20%), Significant (>20%) | Outcomes interviewer blinded? | Intent-to-treat analysis? | If cluster RCT are results properly adjusted? | Authors' judgement of risk of bias | Comment |
| --- | --- | --- | --- | --- | --- | --- | --- | --- | --- | --- |
| Agrasada (2005) | Yes | Yes | Yes | Yes | Moderate (12%) | Unclear | Yes |  | Low |  |
| Aksu (2011) | Yes | Yes | Yes | Yes | Moderate (18%) | Unclear | No – see comments |  | Moderate | 66 mothers were randomized and only 60 in analysis due to drop-out during the intervention. |
| Arifeen (2009) | Yes | Yes | Yes | Yes | NA-See comments | Unclear | Yes | Yes | Low | Two cross-sectional surveys |
| Coutinho (2005) | Yes | Yes | Yes | Yes | Minimal (6%) | Unclear | Yes |  | Low |  |
| Davies-Adetugbo (1997) | Yes | Yes | Yes | Yes | Minimal (5%) | No | Yes |  | Moderate | Outcome assessment performed by peer counsellors |
| Haider (2000) | Yes | Unclear | Unclear | Yes | Moderate (12%) | No | Yes | See Comments | Moderate | Relative risk not adjusted for clustering, risk differences are adjusted |
| Feldens (2007) | Yes | Yes | Yes | Yes | Moderate (14%) | Yes | Yes |  | Low |  |
| Jakobsen (1999) | Yes | Unclear | Yes | No-see comments | Significant (29%) | Unclear | Yes | See Comments | High | Groups differed by ethic group, birth order, sex, and distribution of age at baseline. Authors calculated correction factors for clustering and determined there was no important impact, but data is not presented. |
| Leite (2005) | Yes | Yes | Yes | Yes | Moderate (14%) | Yes | Yes |  | Low |  |
| Morrow (1999) | Yes | Yes | Yes | Yes | Minimal (4%) | No | Yes | See Comments | Low | Crude relative risks not adjusted, GEE results are adjusted |
| Tylleskär (2011) | Yes | Yes | Yes | Yes | Moderate (11%) | Yes | Yes | Yes | Low |  |
