# Supplementary material for: Peer Support and Exclusive Breastfeeding Duration in Low and Middle-Income Countries: A Systematic Review and Meta-Analysis
Source: PLoS One. 2012 Sep 18;7(9):e45143. doi: 10.1371/journal.pone.0045143 (PMC3445598; doi:10.1371/journal.pone.0045143)
Supplement: Table S2 — Studies excluded from the systematic review and meta-analysis. (DOCX) [file pone.0045143.s002.docx]

| **Study ID** | **Reason for Exclusion** |
| --- | --- |
| Aidam 2005 | Counsellors were local nurses and nutritionists |
| Albernaz 2003 | Counsellors were local nurses |
| Bashour 2008 | Counsellors were registered midwives |
| Bhandari 2003 | Counsellors included birth attendants, CHWs, as well as auxiliary midwives and immunization clinic health workers |
| Bhutta 2008 | Intervention and control received counselling |
| Haider 1999 | Counsellors were research physicians |
| Kramer 2001 | Counsellors were healthcare workers |
| Kramer 2007 | Counsellors were healthcare workers |
| Kramer 2008 | Counsellors were healthcare workers |
| Kramer 2009 | Counsellors were healthcare workers |
| Mannan 2008 | Peer counselling not randomized |
| Mattar 2007 | Conducted in Singapore (classified as high income) |
| Ransjo-Arvidson 1998 | Counsellors were midwives |
| Su 2007 | Conducted in Singapore (classified as high income) |
| Susin 2008 | Group counselling led by physician |
| Valdes 2000 | Counsellors were nurses and midwives, also not randomized |
| Vitolo 2008 | Subanalysis of the included Feldens (2007) trial |
| Wong 2007 | Conducted in Hong Kong SAR, China (classified as high income) |
| Zaman 2008 | No data on exclusive breastfeeding |
